# What makes a peer supporter competent? A bottom-up cross-culturally informed multi-method study

**DOI:** 10.1093/geroni/igaf122.2720

**Published:** 2025-12-31

**Authors:** Terry Lum, Stephanie Ming, Yin Wong, Dara Kiu, Yi Leung, Eric Kwok, Edwin Wong, Wai Chi Chan, Gloria Hoi, Yan Wong

**Affiliations:** The University of Hong Kong, Hong Kong, China (People’s Republic); The University of Hong Kong, Hong Kong, China (People’s Republic); The University of Hong Kong, Hong Kong, China (People’s Republic); The University of Hong Kong, Hong Kong, China (People’s Republic); University of Birmingham, England, United Kingdom; The University of Hong Kong, Hong Kong, China (People’s Republic); University of Reading, England, United Kingdom

## Abstract

Peer support is increasingly recognised as an important person-centred means of facilitating mental well-being among older adults. Enhancing competencies among Peer Supporters (PS) and any service provider is crucial to ensuring quality service. Yet, there is limited knowledge concerning what a PS’s core competencies are from the perspectives of multiple key stakeholders and how they could be aptly assessed. We conducted a three-round bottom-up Delphi study involving 30 PS (≥50 years), 14 service users (≥60 years), and 23 social workers/project officers to develop a PS core competency framework. This was followed by constructing a self-rated Peer Supporter Competence Scale (PSCS) for the local population. The psychometric properties of the PSCS were examined in 340 trained PS from a territory-wide community-based stepped-care intervention service for older adults with depressive symptoms in Hong Kong. Thirty-five core competencies were identified with five overarching themes: ‘Self and self-development’, ‘General work ethics’, ‘Work with others’, ‘Work with service users’ (encompassing ‘Interpersonal attitude and skills’, ‘Risk management’, and ‘Recovery facilitation’), and ‘Peer support knowledge’. 14.3% were unique to the local context, 20% had conceptual origins in the West, and 65.7% were shared across cultures. Following standard scale development and validation procedures, the final scale for PS competence assessment comprised 16 items (PSCS-16), demonstrating good reliability (α = 0.84) and convergent validity (r = 0.40 with the General Self-Efficacy Scale, p < 0.001). These findings have implications for enhancing peer support training and demonstrate the feasibility of developing a culturally-informed measure of PS competence using a bottom-up approach.

